# BKT300: A Novel Anti-Leukemic Small Molecule Targeting the Protein Regulator of Cytokinesis 1 (PRC1) Pathway

**DOI:** 10.21203/rs.3.rs-6017610/v1

**Published:** 2025-02-19

**Authors:** Amnon Peled, Michal Abraham, Hanna Wald, Ophir Hay, Shira Hagbi, Lika Gamaev, Jonathan Monin, Gautam Borthakur, Edward Ayoub, Michael Andreeff, Rakefet Rosenfeld, Orly Eizenberg, Arnon Aharon

**Affiliations:** Hadassah University Hospital; AlonBio Ltd; Hadassah University Hospital; Hadassah University Hospital; Hadassah University Hospital; Hadassah University Hospital; The Hebrew University; The University of Texas MD Anderson Cancer Center; The University of Texas MD Anderson Cancer Center; The University of Texas MD Anderson Cancer Center; AlonBio Ltd; AlonBio Ltd; AlonBio Ltd

## Abstract

Protein regulator of cytokinesis 1 (PRC1) is frequently overexpressed in various cancers and is associated with poor prognosis. BKT300 is a small molecule shown to selectively inhibit leukemic cell migration and survival by targeting the PRC1 pathways. The current work aimed to examine the role of PRC1 in acute myeloid leukemia (AML) and to assess the impact of BKT300, a small molecule PRC1 inhibitor, on AML cell viability and tumor growth in mouse xenograft AML models. BKT300 directly bound PRC1, resulting in disrupted actin and microtubule formation, G2/M cell cycle arrest, mitotic catastrophe and apoptosis via the caspase-3 pathway in AML cells. BKT300 inhibited PRC1 dephosphorylation at T481, downregulated CDC25C and upregulated p21, effectively halting the cell cycle and inhibiting leukemic cell proliferation while sparing normal cells. PRC1 was found to be overexpressed in AML patients and cell lines, with high levels associated with reduced overall patient survival. In addition, PRC1 expression levels correlated with BKT300 efficacy. BKT300 treatment led to 98% of tumor growth inhibition and 89.4% of tumor regression in mouse xenograft AML models, without notable impacts on normal hematopoiesis or biochemistry, even at high doses. As a first-in-class targeted therapy, BKT300 presents a promising new treatment option for advanced AML.

## Introduction

In normal cells, the activity of cell-cycle proteins is tightly controlled by their cell-cycle-specific transcription, degradation, dephosphorylation and phosphorylation, by cyclin-dependent kinases (CDKs), or their inhibition thereof, by several CDK inhibitors(1). All these control mechanisms are dysregulated in human cancers, resulting in aberrant activation of cell-cycle-specific proteins. Indeed, genetic lesions within the core cell-cycle machinery, resulting in its hyperactivation, play a causative role in development of most tumor types(2).

Cell cycle inhibition is emerging as a crucial approach in the treatment of acute myeloid leukemia (AML) (3, 4). By targeting key checkpoints, such inhibitors can enhance chemotherapy effectiveness, increase leukemic cell sensitivity to DNA damage, and potentially improve clinical outcomes. Furthermore, they may reduce relapse rates by targeting resistant leukemic stem cells, often linked to treatment failure. Preclinical studies have demonstrated enhanced AML cell death in the presence of cell cycle inhibitors, such as CHK1 and WEE1 inhibitors. Currently ongoing clinical trials are investigating the use of CHK1 inhibitors (5) and WEE1 inhibitors (6) to enhance the cytotoxic effects of standard chemotherapies and improve AML patient responses. Similarly, combinations of cell cycle inhibitors with targeted therapies are being explored to assess their effectiveness in eradicating leukemic stem cells and improving long-term outcomes.

Protein regulator of cytokinesis 1 (PRC1) is a scaffold protein that regulates metabolic pathways that drive cancer cell proliferation, survival and metastasis(7). PRC1 was identified as a microtubule-associated protein and was shown to regulate cytokinesis through microtubule crosslinking (7, 8). The protein is characterized by an N-terminal coiled-coil domain, a central region facilitating microtubule binding and a C-terminal regulatory domain housing a nuclear localization signal(9, 10). PRC1 localizes within the nucleus durin g interphase. Its expression is notably elevated during the S and G2/M phases of the cell cycle and tapers off towards the end of mitosis (10).

PRC1 phosphorylation by cyclin-dependent kinase 1 (CDK1) at T470 and T481 (10) inhibits its oligomerization and microtubule-bundling activity {Jiang, 1998 #77;Mollinari, 2002 #76;Neef, 2007 #48;Zhu, 2006 #88},. During metaphase, phosphorylation of these sites prevents polo-like kinase 1 (PLK1) binding to PRC1, which ensures that PLK1 is only recruited after anaphase onset, when Thr 481 is dephosphorylated (14, 15). Once PLK1 binds PRC1, it phosphorylates it at T602, creating a docking site for stable PLK1-PRC1 binding and enabling PRC1 to target PLK1 to the central spindle (11, 16). The formation of the PRC1-PLK1 complex is crucial for successful cytokinesis and for a shift in PLK1 localization, (11). Moreover, depletion of PRC1 by siRNA disrupts recruitment of PLK1 to the central spindle (11). PRC1 was shown to interact with several other proteins such as p53, Wnt/ß-catenin, CDK16, CCDC69, MgcRacGAP and FAK-paxillin (17, 18, 19, 20, 21, 22).

In vitro and in vivo studies showed that knockdown of PRC1 using siRNA or shRNA significantly inhibited tumor growth and proliferation by arresting cells at G2/M and inducing mitotic catastrophe and apoptosis through the caspase 3 pathway (21, 23, 24). Moreover, its knockdown in human lung cancer cells, hepatocellular carcinoma (HCC) and colon cancer cells inhibited the expression of several key proteins associated with the G2/M phase, such as, cyclin B1, CDC2 and CDC25C, while upregulating p21, p27 and PLK1 (21, 23, 24).

The oncogenic role of PRC1 has been well documented in various solid cancers, including bladder(25), breast(26), liver (27), prostate(28), lung adenocarcinoma (21), gastric(29), colon (24) and ovarian cancers (30). Elevated PRC1 expression in these cancers is associated with aggressive clinicopathological features and poor prognosis, suggesting its potential as both a prognostic indicator and a promising cancer therapy target. Yet, despite its established role in solid tumors, the involvement of PRC1 in AML remains underexplored, with limited studies investigating its function in leukemogenesis.

Efforts have been made to modulate PRC1 activity by targeting upstream kinases or pathways, such as those involving non-estrogen receptor (ER), p53, PLK1 and the Wnt signaling pathway(7). However, to date, no inhibitor specifically targeting PRC1 is available.

**Figure 1:**
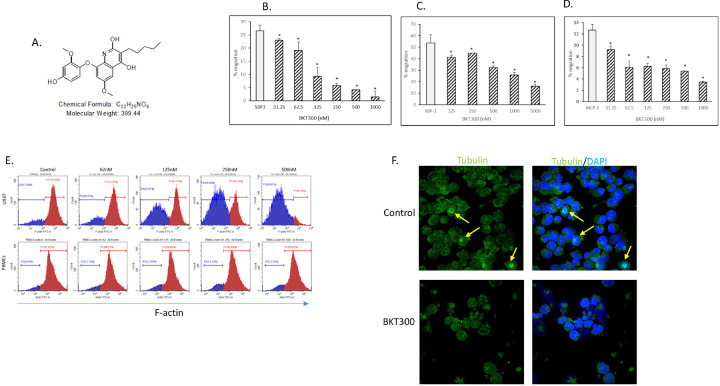
Effects of BKT300 on cell migration, cytoskeletal dynamics and tubulin organization. (A) Chemical structure of BKT300. (B-D) Effect of BKT300 on migration of U937 (B) and Jurkat (C) cells toward CXCL12 and on the migration of THP1 cells toward MCP-1 (D), as measured in an in vitro transwell migration assay. (E) Expression of F-actin in U937 and normal peripheral blood mononuclear cells (PBMCs) following treatment with 62.5– 500 nM BKT300, as measured by flow cytometry following staining with phalloidin. (F) Representative immunofluorescence (IF) images of tubulin staining (green) in U937 cells after 24 h treatment with BKT300 (1000 nM). Yellow arrows indicate mitotic spindles. In B-D, analyses were done in triplicates. Data represent mean ± SD of two independent experiments. * p <0.05 Student’s t-test.

BKT300 (MW 399.33, C_22_H_25_NO_6_) is a small molecule identified through phenotypic screening of a natural compound library designed to discover small molecules that impair AML cell migration and survival. Among the candidates tested, BKT300 stood out for its superior ability to inhibit migration and induce cell death in AML cells. The compound was subsequently optimized through scaffold hopping and structure-activity relationship analyses to enhance its therapeutic efficacy (Fig. 1A). The current study investigated the relevance of PRC1 in AML and examined the AML-targeting capacity of BKT300 as well as its therapeutic potential in preclinical models.

## Materials and Methods

### Hematological cancer Cell lines

Human cancer cell lines, U937 (CVCL_0007), MV4–11 (CVCL_0064), OCI-AML-2 (CVCL_1619), OCI-AML-3 (CVCL_1844), Marimo (CVCL_6992), MOLM-14 (CVCL_7916), THp-1 (CVCL_0006), jurkat (CVCL_0367), K562 (CVCL_0004), NB4 (CVCL_0005), REH (CVCL_1650), HL60 (CVCL_0002), Farage (CVCL_3302), SU-DHL-6 (CVCL_2206), Toledo (CVCL_3611) and SU-DHL-4 (CVCL_0539) were cultured in RPMI 1640 (Gibco, Thermo Fisher, USA), supplemented with 10% fetal calf serum (FCS), 100 U/ml penicillin, 100 μg/ml streptomycin, 2 mM L-glutamine and 1% sodium pyruvate (Biological Industries, Israel). All cells were cultured in a 37 °C, 5% CO_2_ incubator and verified to be mycoplasma-free (Lonza Bioscience, Germany) multiple times throughout the study.

#### Normal cells

Peripheral blood mononuclear cells (PBMCs) were isolated from whole blood using the Ficoll density gradient separation (HISTOPAQUE-1077, Sigma). Monocytes were enriched by adding the RosetteSep Human Monocytes Cocktail (StemCell, cat# 15028), and T cells were enriched by adding the RosetteSep Human T Cell Cocktail (StemCell, cat# 15021). All cells were treated with BKT300 for 24 h. During this period, PBMCs and T cells were activated with αCD3 (30 ng/ml) and IL-2 (1000 IU/ml), while monocytes were activated with lipopolysaccharide (100 ng/ml)

#### Migration of hematological cancer cell lines

Cells were resuspended in RPMI medium (100 μl, 2×10^5 cells/well) and seeded in the upper chambers of Transwell plates (6.5 mm diameter, 5 μm pore size for U937 and Jurkat cells 3421; 8 μm pore size for THP-1 cells 3422, Costar/Corning, ME, USA). The lower chambers were filled with 600 μl RPMI containing 100 ng/ml CXCL12 (PeproTech, 300–28A) or 200 ng/ml MCP-1 (PeproTech, 300–04) and increasing concentrations of BKT300 (31.25–1000 nM). To determine the total cell count (100%), the same number and volume of cells were added to 500 μl RPMI medium and seeded in a lower chamber. Spontaneous migration was assessed in wells containing cells in the upper chamber, but no chemoattractants or BKT300 in the lower chamber. After 3 h, the number of cells in the bottom chamber was quantified using FACScalibur. Migration percentage was calculated as: [(Number of migrated cells - spontaneous migration)/Total number of seeded cells] × 100.

#### Formation of actin filaments

U937 cells or PBMCs (1×10^6/ml) were incubated with BKT300 (62.5–500 nM) for 24 h. Cells were then washed and re-suspended in 200 μl Fix/Perm buffer (eBioscience, 00-5123-43), and incubated for 20 min, at 4 °C. After washing the cells with Perm/Wash buffer (1 ml) (eBioscience, 00-8333-56), phalloidin (Thermo Fisher Scientific, A12379) was added (2.5 μl in 100 μl Perm/Wash) for 20 min, at 4°C, after which, cells were washed with PBS and phalloidin fluorescence was analyzed by a flow cytometer (CytoFLEX flow cytometer (Beckman Coulter, Krefeld, Germany).

#### Formation and organization of microtubules

U937 cells (1×10^6 cells/ml) were cultured in RPMI on round cover-slides in 12-well plates. After 24 h, the medium was changed and supplemented with BKT300 (1 μM), for 24 h. Cells were then washed with PBS (2 ml/well), fixed by adding 2 ml Fixation Buffer (eBioscience, 00–8222-49) for 30 min, and then permeated with 2 ml Permeabilization Buffer (eBioscience, 00-8333-56), for 30 min. The cells were then washed with PBS, blocked with 10% normal donkey serum in PBS-Tween for 1 h, at room temperature (RT), and then incubated with anti-tubulin Alexa Fluor^®^ 488 antibody (Abcam, ab195887) diluted in PBS-Tween (1:2000), for 1 h, at RT, followed by the addition of DAPI (1:1000) (Sigma-Aldrich, D9542). The cells were washed three times with PBS-Tween, and slides were mounted using EMS mounting (Fluoromount-G^™^, Invitrogen, Waltham, MA, USA). After drying in the dark, fluorescence was measured with a confocal laser scanning biological microscope- (OLYMPUS, FLUOVIEW FV1000).

### Tubulin polymerization assay

Tubulin polymerization was assessed with the Tubulin Polymerization Assay Kit (Cytoskeleton, BK006P), per the manufacturer’s instructions. The effect of BKT300 (100 nM or 1000 nM) was tested. As positive control we used Taxol (10 μM), which is a polymerization enhancer. All treatments were tested in triplicates. The optical density (OD) was measured at 340 nm, in a microplate reader in kinetic mode every minute, for at least 1 h, at 37°C, in a chamber protected from light.

#### Cell cycle analysis

Cells (1×10^6 cells/ ml) were cultured in 12-well plates with BKT300 (7.8–1000 nM), for 24 h. Cells were then harvested and washed with PBS. The pellet was fixed with 200 μl Fix/Perm buffer (eBioscience, 00-5223-56), for 20 min, at 4 °C, and then washed with 1 ml Perm/Wash buffer (eBioscience, 00-8333-56). Following centrifugation, the cells were resuspended in 100 μl Perm/Wash supplemented with 4 μl 7-aminoactinomycin D (7-AAD) (Sigma-Aldrich, A9400) and incubated, in the dark, for 20 min, at 4 °C, before 300 μl PBS were added. Cells were analyzed by flow cytometry, with 20,000 events collected per sample. The distribution of cells in the major phases of the cycle (G0/G1, G2/M and Sub-G0 (dead cells)) was determined and IC_50_ was calculated using GraphPad Prism software.

#### Apoptosis assay

Apoptosis was determined using an annexin-V kit (eBioscience, 88-8005-74). Cells (1×10^6 cells/well) were incubated in 24-well plates, with BKT300 added, for 24 h, at the indicated concentrations. Cells were then collected, centrifuged and stained with Annexin-V and propidium iodide (PI), according to manufacturer’s instructions. The number of viable cells (annexin-V-negative/PI-negative) and apoptotic cells (annexin-V-positive) were determined by flow cytometry (FACS). IC_50_ was calculated using GraphPad Prism software.

### Western blot (WB) analysis

Cells (1×10^6 cells/ml) were cultured with BKT300, in 12-well plates, for 24 h. Protein extracts were prepared by incubating cells in lysis buffer (Cell Signaling, 9803) supplemented with a protease/phosphatase inhibitor cocktail (1:100; Cell Signaling, 5872) and 100 mM phenylmethylsulfonyl fluoride (Cell Signaling, 8553) (100 μl per 1×10^7 cells), for 30 min, at 4 °C. Protein concentration was quantified using the total protein Bradford assay. Samples were denatured by boiling cell lysates in sample buffer, at 95 °C, for 5 min. Equal amounts of protein (30 μg) were loaded onto a 10% SDS-PAGE gel, along with a molecular weight marker. The gels were run in MOPS running buffer (A2S technologies) for 65 min, at 140 V. The proteins were transferred from the gel to a nitrocellulose membrane, which was then washed with PBS-T (PBS + 0.5% Tween-20), for 5 min, and blocked with 5% bovine serum albumin (BSA), for 1 h. The membrane was then washed twice with PBS-T, for 5 min each. Protein bands were identified by incubating membranes with the relevant primary antibody in TBS-T, overnight, with shaking. The membrane was then washed twice with PBS-T for 5 min, incubated with horseradish peroxidase (HRP)-conjugated anti-mouse (Dako, K4001) or anti-rabbit (Dako, K4003) antibodies, for 1 h, and then washed 3 times with TBS-T, for 5 min each. Blotted protein bands were visualized using the EZ-ECL Kit (Biological Industries, 20-500-500), per the manufacturer’s instructions, and a ChemiDoc MP imaging system. Protein band intensity was quantified by computerized densitometry using Image Lab software. The same blots were stripped and re-probed with anti-human β-actin antibody (Cell Signaling, 8457) and results were normalized to actin expression.

### Cell synchronization with RO-3306

U937 cells (1×10^6 cells/ml) were incubated with RO-3306 (9 μM) (Sigma, SML0569) in RPMI medium, for 20 h. Cells were then washed twice with medium, and then incubated in medium with increasing concentrations of BKT300 (6.25, 12.5, 25, 50 and 100 nM), for 4 h. Cells were then harvested and washed with PBS and cells cycle analysis was performed.

### Screening for the enzymatic activity of 376 kinases

In vitro profiling of a 376-kinase panel was performed at Reaction Biology Corporation (www.reactionbiology.com, Malvern, PA) using the “HotSpot” assay platform, which measures kinase activity based on the incorporation of radioactive 33P-ATP into substrate peptides, with results expressed as percent remaining activity and IC50 values determined through curve fitting. BKT300 10 μM was tested in single-dose duplicate mode. The control compound, Staurosporine, was tested in 10-dose IC50 mode, with 4-fold serial dilutions, starting at 20 μM or 100 μM.

### KINOMEscan

The interaction between BKT300 (1, 5 and 10 μM) and seven kinases implicated in cell cycle arrest, i.e., PLK1, AURKA, AURKB, BUB1, CHEK2, CIT and TTK, was tested by DiscoverX Eurofins Corporation (https://www.discoverx.com/). The KINOMEscan^™^ platform, utilized by DiscoverX, employs a novel and proprietary active site-directed competition binding assay to quantitatively measure interactions between test compounds and kinases.

### Immunohistochemistry

Paraffin-embedded tumor sections were subjected to dewaxing, rehydration and antigen retrieval in EDTA buffer (pH 8 for CDC25C staining; pH 9 for total PRC1 staining), at 84 °C, for 20 min. After treatment with H_2_O_2_ and washing with PBS-T, CAS blocking reagent (Zymed Laboratories, San Francisco, CA, USA) was applied. For CDC25Cstaining, sections were then incubated, overnight, at 4 °C, with anti-human CDC25C antibody (1:500; Abcam, ab32444), and then with rabbit HRP-conjugated antibody for 30 min, at RT. For total PRC1 staining, sections were incubated, overnight, at 4 °C, with anti-human total PRC1 antibody (1:200; Sigma, HPA034521) and then with biotinylated goat anti-rabbit antibody, for 30 min, at RT, followed by streptavidin-HRP treatment. Color was developed using AEC substrate and samples were counterstained with hematoxylin.

For pPRC1 staining, sections were dewaxed, rehydrated, and subjected to antigen retrieval in citrate buffer (pH 6), at 84 °C, for 20 min. After cooling, samples were blocked with 10% normal horse serum containing 0.3% Triton X-100, for 1 h. Sections were then incubated, overnight, at 4 °C, with anti-human phosphorylated pPRC1 (pPRC1) antibody (1:100; Abcam, ab62366). Following PBST wash, H_2_O_2_ treatment, and incubation with anti-rabbit HRP-conjugated antibody, for 30 min, RT, color was developed using AEC substrate, and sections were counterstained with hematoxylin.

Positively and negatively stained cells in 8–10 fields per tumor sample were manually counted. The percentage of positive staining was calculated for each field, and the overall average number of positively stained cells was determined for each individual tumor.

### Intracellular fluorescence staining for pPRC1 and PRC1

Cells (1×10^6 cells/ ml) were cultured, for 24 h, in 96-well plates in RPMI medium supplemented with BKT300 (31.2, 62.5, 125, 250, 500 nM) and then fixed by incubating them, for 20 min, at RT, in an equal volume of IC Fixation Buffer. Following centrifugation (500 × g, 5 min, RT), the cells were resuspended and incubated (30 min, 4 °C) in 1 ml ice-cold 100% methanol, and then washed with 2 ml Flow Cytometry Staining Buffer (eBioscience, 00-4222-26). Following centrifugation (500 × g for 5 min, RT) and aspiration of the supernatant, the cells were resuspended and incubated with anti-pPRC1 antibody or anti-PRC1 antibody (1:50; Abcam, ab51248) diluted 1:50 in 100 μl Flow Cytometry Staining Buffer, for 30 min, at RT, protected from light. Cells were then washed with 2 ml Flow Cytometry Staining Buffer, centrifuged (500 × g, 5 min, RT), and incubated with goat anti-rabbit IgG FITC (Abcam, AB97050) diluted 1:50 in 100 μl Flow Cytometry Staining Buffer (30 min, RT, protected from light). After washing the cells with 2 ml Flow Cytometry Staining Buffer, and centrifugation (500 × g, 5 min, RT), the cells were resuspended in Flow Cytometry Staining Buffer and analyzed by flow cytometry and by IncuCyte.-a real-time, quantitative live-cell analysis IncuCyte ZOOM^™^ apparatus. Data processing and analysis were performed using the IncuCyte S3 Live-cells Analysis System (Essen Bioscience, Michigan, USA).

### ELISA for PRC1

A 96-well plate was coated with recombinant human PRC1 (Creative BioMart, PRC1–527H; 1 μg/well/100 μl) in 0.1 M carbonate, pH 9.5, and incubated overnight, at 4 °C. Some wells were precoated with BSA. After incubation, 200 μl blocking buffer (PBSx1 + 4% BSA) were added to each well, for 1 h, at RT. BKT300–3-C5–4-PEG4-biotin or BKT300–3-C5–1b-PEG4-biotin (10, 50 or 100 μg/well; 100 μl/well) was then added to each well, for 2 h, RT. Positive controls were incubated with anti-PRC1 instead. Human streptavidin-HRP (1:10,000; Peprotech, HRP-4UL) was added to wells with BKT300–3-C5–4-PEG4-biotin and BKT300–3-C5–1b-PEG4-biotin, while anti-rabbit-HRP (1:50; Dako, K4003) was added to wells with anti-PRC1. The plate was incubated at RT, for 2 h. Subsequently, 100 μl TMB substrate solution (SouthernBiotech, 0412–01) were added to each well and incubated at RT, for 20 min. Then, 50 μl stop solution (SouthernBiotech, 0410–01) were added, and color development was monitored at 450 nm using an ELISA plate reader.

### MicroScale thermophoresis (MST)

PRC-1 (Creative BioMart, PRC1–527H) was labeled with the Monolith NT^™^ Protein Labeling Kit RED-NHS dye at a molar ratio of 1:3, at RT, for 30 min, in the dark, according to the manufacturer’s instructions. The labeled PRC-1 was then diluted 1:40 with PBSx1.

BKT300 was initially dissolved in DMSO (100 mM) and subsequently diluted 1:322 with PBSx1, to a final concentration of 310 μM. The A1 molecule, an analog of BKT300 with no discernible biological activity, served as a negative control.

The BKT300 and A1 solutions were serially diluted with PBS containing DMSO and mixed 1:1 with 155 μM BKT300 or A1 (155,000 nM). To avoid buffer effects, the dilution buffer was prepared so that the final DMSO concentration was the same in all samples. The samples were then loaded into capillaries and analyzed with a Monolith NT.115 instrument (NanoTemper Technologies). The assay was carried out with 40% LED power and 80% MST power. Signal Thermophoresis + T-Jump Data were used to calculate the dissociation constant (Kd). Data were analyzed using software NT Analysis and Origin9.

### Surface plasmon resonance (SPR)

The interaction between recombinant PRC1 and BKT300 was investigated using surface plasmon resonance (SPR) spectroscopy performed with a Biacore 3000^®^ instrument. PRC1 was immobilized on the sensor chip surface according to the manufacturer’s instructions. BKT300 was prepared in a series of five serial 1:2 dilutions in HBS-E buffer, to concentrations of 1.00e-5 M, 5.00e-6 M, 2.50e-6 M, 1.25e-6 M, and 6.00e-7 M. The SPR experiments were conducted at a flow rate of 10 μL/min, with an injected analyte (BKT300) volume of 40 μL and a dissociation time of 60 s (KINJET 40/60). During analyte injection, the interaction between BKT300 and immobilized PRC1 was monitored in real-time, with the response measured in refraction units (RU) and recorded as a sensorgram. Following analyte injection, a running buffer (HBS-E) was injected to facilitate dissociation of the bound BKT300 from the immobilized PRC1, allowing for the calculation of the dissociation constant (K_D). To remove any residual analyte, the chip surface was regenerated with a regeneration buffer (10 mM HCl). Kinetic parameters, including the association constant (K_A) and dissociation constant (K_D), were derived from the sensorgram data using optimized fitting methods. SPR was performed by Inoviem Scientific (https://www.inoviem.com/Bioparc 2–850, Boulevard Sébastien Brant 67400 Illkirch-Graffenstaden – France).

### In Vivo Experiments

#### Mice

Xenograft experiments were carried out in 6–8-week-old NOD scid gamma (NSG) mice (Jackson Laboratory, Bar Harbor, Maine, USA) that were maintained under defined flora conditions at the Hebrew University pathogen-free animal facility (Jerusalem, Israel). All experiments were approved by the Institutional Animal Care and Use Committee (IACUC) of the Hebrew University (Ethics permits: MD-21-16482-4, MD-16-14753-4, MD-21-16481-5) and were performed according to the guidelines for animal experimentation and welfare of the Authority for Biological and Biomedical Models at the Hebrew University.

#### AML U937 xenograft model

U937 cells (10×10^6 cells/mouse) were subcutaneously (SC) injected into the right flank of NSG mice in a final volume of 200 μL. Mice (n = 7/group) were randomly divided into groups to receive BKT300 injections once daily at 2.5 mg/mouse, from days 3 to 7 post-U937 cell injection (early administration), or twice daily at 2.5 mg/mouse, from days 10 to 12 post-U937 cell injection (late administration). Tumor size was measured with a caliper, and tumor volume was calculated using the formula: Tumor volume = widtĥ2 * length/2. Mice were euthanized 24 h after the final treatment, and tumors were excised, weighed, and stained to assess tissue damage.

Tumor growth inhibition (TGI) was calculated using the formula 100 - [ΔT/ΔC * 100], where ΔT is the volume change for treated tumors, and ΔC is the volume change for control tumors.

Tumor growth regression (TR) was calculated using initial (i) and final (f) tumor measurements for the treatment (T) group by the formula: %TR = [1 − (Tf/Ti)] × 100.

#### Preparation of BKT300

BKT300 was dissolved to a concentration of 30 mg/mL in a 50:50 (v/v) mixture of Cremophore EL and ethanol. Prior to administration, the solution was further diluted 1:6 with saline to a final concentration of 5 mg/mL. For SC administration, 1 mg (0.2 mL), 1.5 mg (0.3 mL), 0.4 mL (2 mg), or 2.5 mg (0.5 mL) BKT300 were injected. Control mice were treated SC with vehicle consisting of the same 50:50 (v/v) mixture of Cremophore EL and ethanol, which was diluted 1:6 with saline.

#### The effect of BKT300 on hematopoiesis and blood chemistry

NSG mice were randomly assigned to three groups three days after SC injection of U937 cells, to receive either no treatment (control; n = 2), vehicle (n = 4) or BKT300 (n = 6). The BKT300-treated mice received a daily dose of 2.5 mg from days 3 to 7 and again from days 10 to 14. Blood samples were collected on day 17 and sent to American Medical Laboratories in Herzliya, Israel (https://www.aml.co.il/en/), for complete blood count analysis. Additionally, a comprehensive chemistry panel in collected serum was assessed.

#### AML xenograft model of MV4–11 cells

An AML model was generated by irradiating NSG mice at 200 RAD on day 0, followed by intravenous (IV) injection of 10×10^6 MV4–11 cells on day 1. BKT300 (2.5 mg) was SC administered on days 12–16 and 19–20. Mice were sacrificed 24 h after the last treatment (day 21), and blood and bone marrow (BM) samples were collected to assess AML cell levels by FACS, after staining with a fluorescent antibody against human CD45.

#### Bioinformatics analysis and data availability

Expression data collected from 14 cancer types in TCGA PanCancer studies were downloaded from cBioPortal for Cancer Genomics, cBioPortal (30, 31, 32). Expression levels from cBioPortal are based on data generated by the TCGA Research Network https://www.cancer.gov/tcga,. The expression level analysis was limited to cancer types with at least 500 samples. Expression comparisons were based on batch normalized RNA-seq by expectation maximization (RSEM) values.

In addition, generated by the Therapeutically Applicable Research to Generate Effective Treatments (https://www.cancer.gov/ccg/research/genome-sequencing/target) initiative, phs000465 (AML), and available at NCI Genomic Data Commons (GDC)(https://portal.gdc.cancer.gov) (33)were downloaded for analysis.

PRC1 and CDC25C expression levels, in transcripts per million (TPM) units, of 3172 patientswere downloaded from GDC. Patient data were categorized as either primary or recurrent cancer and for each cancer type, as either bone marrow or peripheral blood tissue source.

Survival data were downloaded from the GDCTARGET-AML project after applying the GDC clinical data analysis tool. Data were categorized by cancer type (primary or recurrent) and by tissue source (bone marrow or peripheral blood). Expression levels for each gene of interest were ranked from high to low and split to top ranked, mid-ranked and low ranked, with a third of the total samples in each slice.

### Statistical analysis

Statistical analyses were performed utilizing GraphPad (RRID:SCR_002798) or Excel. p values were calculated using an unpaired two-tailed Student’s t-test. p ⩽ 0.05 was considered statistically significant.

## Results

### BKT300 selectively inhibits migration of leukemic cells

Migration assays showed that BKT300 significantly inhibited the migration of leukemic cancer cell lines, including AML U937 cells (Fig. 1B) and acute T cell leukemia Jurkat cells (Fig. 1C), toward CXCL12. Moreover, BKT300 inhibited the migration of AML THP-1 cells toward monocyte chemotactic protein-1 (MCP-1) (Fig. 1D).

### BKT300 inhibits the formation and organization of actin filaments and microtubules

Microtubules and actin filaments are crucial elements of the internal cellular framework involved in cell migration and division (35). BKT300 significantly inhibited the formation of actin filaments in a dose-dependent manner, as demonstrated in U937 cells (Fig. 1E). No discernible inhibitory effect of BKT300 on the expression of actin filaments was noted in normal peripheral blood mononuclear cells (PBMCs), implying its cancer cell-specific activity (Fig. 1E). Fluorescence microscopy analyses identified microtubule filaments (Fig. 1F) in untreated U937 cells, including organization of microtubules into mitotic spindles, indicating normal cell division (Fig. 1F). In contrast, after treatment with BKT300, no dividing cells were observed, and neither the organization nor formation of microtubules was evident. BKT300 had neither an enhancing nor an inhibiting effect on tubulin polymerization (data not shown). These findings suggest that BKT300 interferes with the formation and organization of microtubules, thereby inhibiting the formation of mitotic spindles in AML cells.

#### BKT300 arrests cancer cells at G2/M and induces apoptotic cell death through the caspase 3 pathway

**Figure 2:**
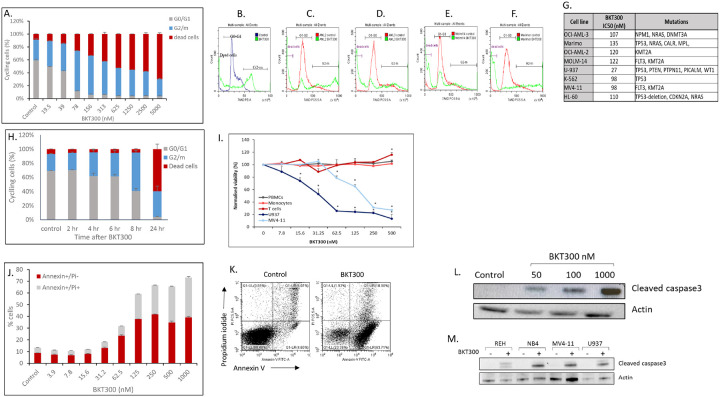
BKT300 arrests cancer cells at G2/M and induces apoptotic cell death through the caspase 3 pathway (A) 7-AAD cell cycle analysis of U937 cells after 24 h of treatment with BKT300 at the indicated doses. (B) Representative fluorescence-activated cell sorting (FACS) histogram of 7-AAD cell cycle analysis for untreated and BKT300-treated (62.5 nM, 24 h) U937 cells. (C-F) Representative FACS histograms of 7-AAD cell cycle analysis for untreated control and BKT300-treated (250 nM; 24 h) OCI-AML-2 (C), OCI-AML-3 (D), Molm14 (E) and Marimo (F) cell lines. (G) IC_50_ values, calculated using GraphPad for each cell line, as determined from 7-AAD cell cycle analysis. (H) 7-AAD cell cycle analysis of U937 cells at the indicated time points following treatment with 62.5 nM BKT300. (I) Normalized viability of U937, MV4–11, normal peripheral blood mononuclear cells (PBMCs), normal monocytes and normal T cells after 24 h of treatment with BKT300 at the indicated doses, as measured by FACS following propidium iodide (PI) staining. During the 24-h treatment period, normal PBMCs and T cells were activated with αCD3 (30 ng/ml) and IL-2 (1000 IU/ml), while monocytes were activated with lipopolysaccharide (100 ng/ml). (J) FACS analysis of proportions of Annexin/PI-positive U937 cells treated for 24 h with BKT300 at the indicated doses. (K) Representative flow cytometry results of U937 cells stained with annexin/PI after a 24-h treatment with 125 nM BKT300. (L) Western blots for cleaved caspase-3 in U937 cells treated with 50, 100, or 1000 nM BKT300, for 24 h. (M) Western blots for cleaved caspase-3 in different leukemic cell lines (REH, NB4, MV4–11 and U937 cells) after treatment with 1000 nM BKT300, for 24 h. In A-J, analyses were performed in triplicates. Data represent mean ± SD of three independent experiments. * p <0.05 Student’s t-test.

Further analysis of the effects of BKT300 on the cell cycle revealed that the compound induces cell accumulation in the G2/M phase, accompanied by a reduction in the number of cells in the G0/G1 phase. In addition, U937 cell death was induced, with IC_50_ measuring 27 nM (Fig. 2A–B). Observations were similar in other AML cell lines, including MV4–11, AML2, AML3, Molm14 and Marimor (Fig. 2C–G), despite the different mutations carried by each line, including mutations in p53 (Fig. 2G). The effects of BKT300 on U937 cells were mild 4 h after exposure, and manifested by fewer cells in G0/G1, alongside a rise in the number of cells in the G2/M phases. The effect intensified by 6 h and 8 h post-treatment, while significant cell death was only observed after 24 h (Fig. 2H).

After a 24-h exposure, an increase in the number of apoptotic annexin-positive cells as compared to baseline was measured (Fig. 2J and 2K), alongside a dose-dependent increase in cleaved caspase-3 levels in U937 cells (Fig. 2L) as well as in several other hematological malignancy cell lines, including REH, NB4 and MV4–11 (Fig. 2M). While BKT300 reduced viability of U937 and MV4–11 AML cell lines, it had no impact on the viability of normal PBMCs, monocytes, or T cells (Fig. 2I). In fact, 500 nM BKT300 even led to an increase in T cell counts.

### BKT300 downregulates the expression of CDC25C but not CDC2 and upregulates p21

**Figure 3:**
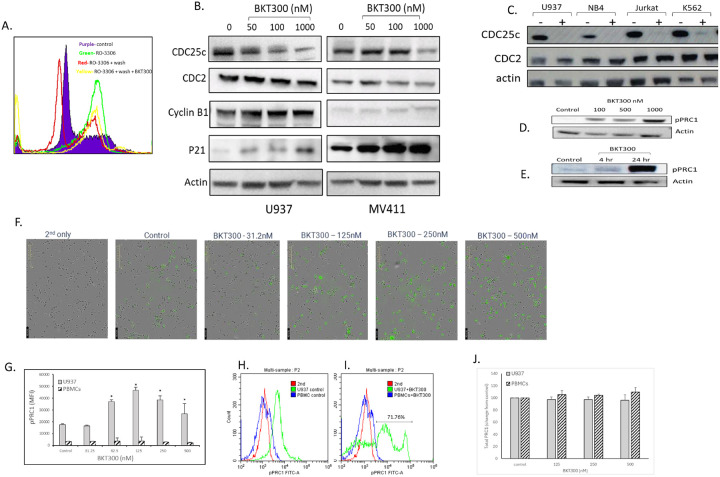
BKT300 downregulates CDC25C without affecting CDC2, upregulates p21 and arrests PRC1 in a phosphorylated state. (A) 7-AAD cell cycle analysis of U937 cells after incubation with 9 mM RO-3306, for 20 h (green), incubated with RO-3306 for 20 h followed by a 4-h washout period (red), or incubated with RO-3306 followed by a 4-h washout period and subsequent addition of 1000 nM BKT300 (yellow). Control cells were left untreated (purple). (B) Western blots for CDC25C, CDC2, cyclin B1 and p21 in U937 and MV4–11 cells following 24 h treatment with 50, 100, or 1000 nM BKT300. (C) Western blots for CDC25C and CDC2 in U937, NB4, Jurkat and K562 cells following 24 h treatment with 1000 nM BKT300. (D) Western blots for pPRC1 (T481) in U937 cells after 24 h incubation with 100, 500, or 1000 nM BKT300. (E) Western blots for pPRC1 (T481) in U937 cells after 4 h or 24 h incubation with 1000 nM BKT300. (F) U937 cells were treated for 24 h with 31.25, 125, 250, or 500 nM BKT300 and then stained with a fluorescent antibody targeting pPRC1 (T481). Images were captured using the IncuCyte system. (G) Mean fluorescence intensity (MFI) of pPRC1 in U937 cells and normal peripheral blood mononuclear cells (PBMCs) following 24 h treatment with 31.25, 62.5, 125, or 500 nM BKT300, as measured by flow cytometry. (H-I) Representative fluorescence-activated cell sorting histograms showing the expression of pPRC1 in control untreated U937 cells and PBMCs (H), and after a 24 h treatment with 125nM BKT300 (I). (J) Effect of 24 h treatment with 125, 250 and 500 nM BKT300 on the expression of total PRC1 in U937 cells and normal PBMCs, shown as the change from control levels, as measured by flow cytometry. Analyses were performed in triplicates. Data represent mean ± SD of two independent experiments. * p <0.05 Student’s t-test.

To explore the mechanism of action of BKT300, the reversible CDK1 inhibitor RO-3306 was used to synchronize cells in the G2/M phase without triggering apoptosis. After RO-3306 was washed out, the cells exited the arrest and returned to the G0/G1 phase within 4 h. However, addition of BKT300 post-washout prevented cell from returning to G0/G1; cells remained in the G2/M phase and underwent apoptosis (Fig. 3A). This suggests that BKT300 targets a protein involved in G2/M arrest and apoptosis. Indeed, BKT300 treatment led to downregulation of CDC25C expression in U937 and MV4–11 cells, as well as in other leukemic cell lines (Fig. 3C), without affecting its enzymatic activity (Supplementary Data, Figure S1) or the expression of CDC2 and cyclin-B1. BKT300 also increased p21 expression in both cell lines (Fig. 3B). No effects on CDC2 expression (Fig. 3C), on the enzymatic activities of CDC25C, CDC25A, or CDC25B, or on a panel of 376 kinases (data not shown) were noted. Additionally, no interaction between BKT300 and key kinases involved in cell cycle arrest, including PLK1, AURKA, AURKB, BUB1, CHEK2, CIT, and TTK, was detected (data not shown).

### BKT300 arrests PRC1 in a phosphorylated state at position T481

Treatment of U937 cells with BKT300 resulted in a dose-dependent increase in PRC1 phosphorylation at Thr 481 (Fig. 3D). This increase was observed within 4 h, and peaked at 24 h (Fig. 3E). The elevation in pPRC1 levels following BKT300 treatment was further confirmed using the Incucyte system (Fig. 3F). FACS analysis also demonstrated a dose-dependent rise in pPRC1 levels in BKT300-treated U937 cells, while no effect on pPRC1 levels was observed in normal PBMCs (Fig. 3G–I). Notably, BKT300 did not impact overall PRC1 levels in U937 cells, or in normal PBMCs (Fig. 3J).

#### Elevated PRC1 levels in AML correlates with CDC25C expression, sensitivity to BKT300, and impact on patient survival

**Figure 4:**
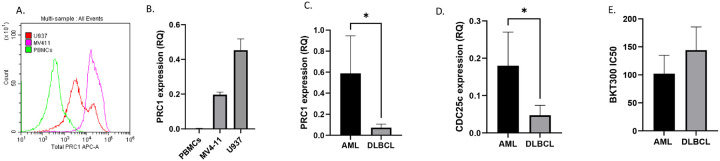
PRC1 and CDC25C expression levels and BKT300 efficacy in AML cell lines (A) Flow cytometry histograms showing baseline levels of total PRC1 in U937, MV4–11 and normal peripheral blood mononuclear cells (PBMCs). (B) Average PRC1 RNA expression levels in U937, MV4–11 and normal PBMCs, as measured by real-time PCR. (C) Average PRC1 RNA expression levels across AML cell lines (n=9) and DLBCL cell lines (n=4). *p=0.0182 (D) Average CDC25C RNA expression levels across AML cell lines (n=9) and DLBCL cell lines (n=4). *p=0.019 (E) Average IC_50_ of BKT300 in AML cell lines (n=9) and DLBCL cell lines (n=4). p = 0.08 Data represent mean ± SD. * p <0.05 Student’s t-test.

**Figure 5:**
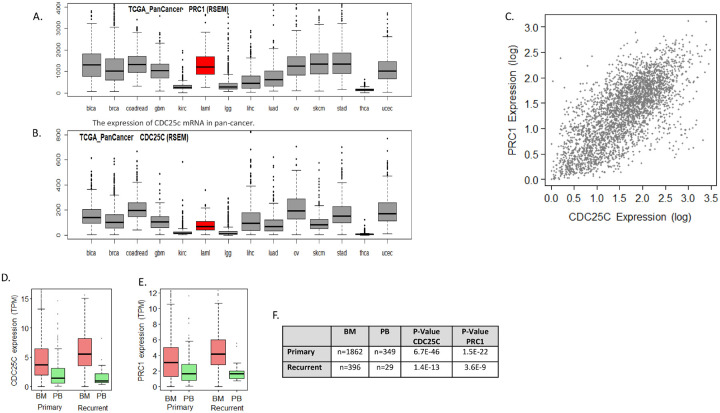
Bioinformatic analysis of PRC1 and CDC25C expression, mutation correlations, and prognostic significance in AML (A, B) Distribution of PRC1 (A) and CDC25C (B) gene expression levels across 14 cancer types. Batch-normalized RSEM values are plotted on the Y-axis and displayed as box plots. Cancer types on the X-axis are abbreviated as follows: BCLA - bladder urothelial carcinoma, BRCA - breast invasive carcinoma, COADREAD - colon and rectum adenocarcinoma, GBM - glioblastoma multiforme, KIRC - kidney renal clear cell carcinoma, LAML - acute myeloid leukemia, LGG - low-grade glioma, LIHC - liver hepatocellular carcinoma, LUAD - lung adenocarcinoma, OV - ovarian serous cystadenocarcinoma, SKCM - skin cutaneous melanoma, STAD - stomach adenocarcinoma, THCA - thyroid carcinoma, UCEC - uterine corpus endometrial carcinoma. (C) Scatter plot illustrating the correlation between CDC25C expression levels and PRC1 expression levels of 3172 AML patients. Data were downloaded from GDC TARGET-AML project. Each dot represents one sample (N=3172). The calculated Pearson correlation is 0.704 (p-value < 1E-200). This same expression data was also employed in (D, E). Box plots demonstrating expression level distribution of PRC1 (D) and CDC25C (E) for primary and for recurrent cancer samples derived from bone marrow (BM) or peripheral blood (PB). (F) A summary table for figures D and E indicating the number of patients in each category and p value assessing the similarity between BM and PB groups. p value was computed using the Kruskal-Wallis test. (G-J) Gene expression levels of PRC1 (G, I) and CDC25C (H, J) in patients carrying common AML-associated gene mutations. Gene expression levels are presented as batch-normalized RNA-seq by expectation maximization (RSEM) values (G-H), or as log2-transformed reads per kilobase per million mapped reads (RPKM) values (I-J). (K-P) Kaplan-Meier survival curves of patients with varying expression levels of PRC1 (K-M) or of CDC25C (N-P). Results are shown for three sample sources: peripheral blood from primary cancers (K, N), recurrent cancers (L, O), and BM from primary cancers (M, P).

Normal PBMCs exhibited significantly lower levels of PRC1 compared to U937 and MV4–11 cells, both at the protein (Fig. 4A) and RNA (Fig. 4B) levels. Similarly, PRC1 and CDC25C mRNA levels in various AML cell lines were significantly higher compared to lymphoma cell lines (Fig. 4C–D). Additionally, AML cell lines displayed greater sensitivity to BKT300 (Fig. 4E). This aligned with the overexpression of PRC1 in AML patients (Fig. 5A), with a noted correlation between the expression of PRC1 and CDC25C (Fig. 5B–C). Of note, the levels of both PRC1 and CDC25C in the BM were higher than those observed in the peripheral blood of AML patients (Figs. 5D–E). When considering AML subpopulations characterized by established mutations, AML patients with TP53 mutations exhibited relatively high levels of PRC1 expression (Fig. 5G and 5I). Overall survival data extracted from GDC - TCGA, showed a correlation between low expression of both PRC1 and CDC25C and better survival outcomes (Fig. 5K–P).

### BKT300 efficiently and specifically binds PRC1

**Figure 6:**
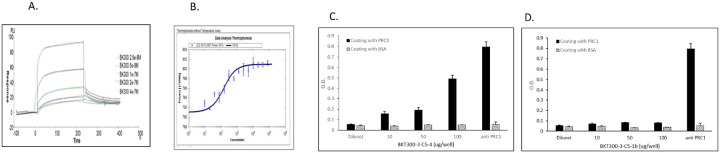
BKT300 efficiently and specifically binds PRC1 (A) Sensorgram showing the interaction between BKT300 and recombinant PRC1, as measured by surface plasmon resonance (SPR). (B) Binding curve determined using the MicroScale Thermophoresis (MST) assay, showing the interaction between BKT300 and PRC1. (C-D) ELISA results, presented as optical density (OD) values, illustrating the binding of the active BKT300–3-C5–4 analog (C) and the inactive BKT300-3-c5-1b analog (D) to PRC1, with nonspecific binding to BSA used as a control.

The interaction between BKT300 and PRC1 was evaluated in multiple assays. SPR spectroscopy revealed a high affinity of BKT300 for PRC1, with a K_d_ of 2.83e-08 M (Fig. 6A), while no binding was observed between BKT300 and CDC25C or DCAF11. Similarly, the inactive analog BKT300-A1 did not bind PRC1. MST confirmed this interaction, showing a K_d_ of 104 ± 12.2 nM for PRC1 (Fig. 6B), with BKT300-A1 again showing no binding. ELISA assays showed specific, dose-dependent binding of BKT300-C5–4 + biotin (active) (Fig. 6C), but not of BKT300–3-C5–1b + biotin (inactive) (Fig. 6D) to PRC1. These results confirm that BKT300 specifically binds to PRC1, and its biological activity appears to depend on this interaction.

### BKT300 inhibits tumor growth and induces tumor regression in AML xenograft mouse models

**Figure 7:**
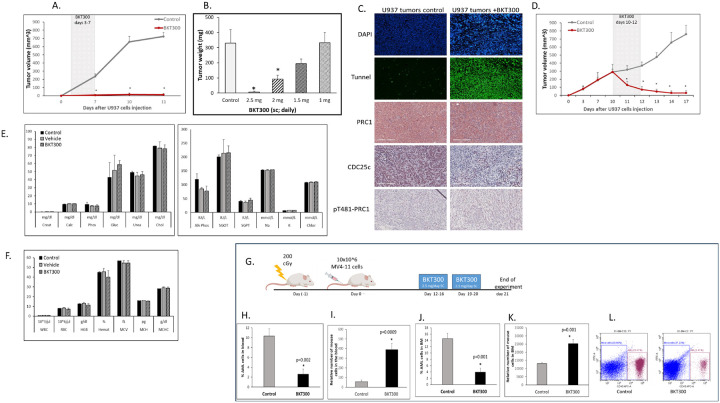
BKT300 inhibits tumor growth and induces tumor regression in xenograft mouse models of AML (A) Average tumor volume over time in NSG mice bearing a subcutaneous human AML U937 cell tumor and treated with 2.5 mg BKT300, once daily, on days 3–7 (n=7). (B) Tumor volume in NSG mice bearing a subcutaneous human AML U937 cell tumor and treated once daily with 1, 1.5, 2, or 2.5 mg/day BKT300, on days 3–7. Tumor weight was measured 24 h after the last treatment (n=7). (C) Representative images of U937 tumor tissue extracted from untreated animals and from animals treated with BKT300, as described in panel A, showing DAPI/TUNEL staining for apoptotic cell death and immunohistochemical stains for total PRC1, CDC25C and phosphorylated PRC1 (pPRC1). Scale bar: 200 μm. (D) Average tumor volume over time in NSG mice bearing a subcutaneous human AML U937 cell tumor and treated with 2.5 mg BKT300, twice a day on days 10–12 (n=7). (E-F) Complete blood count (E) and blood chemistry (F) of untreated (n=2), vehicle-treated (n=4), or BKT300-treated mice (n=6) (2.5 mg once daily on days 3–7 and days 10–14) bearing subcutaneous U937 tumors. Blood samples were collected 72 hours after the last treatment. (G) Experimental schema for in vivo efficacy assessment of BKT300 in an intravenously established human MV4–11 xenograft model in NSG mice. (H) Average percentage of human MV4–11 AML cells in the blood of untreated control mice and mice treated with BKT300 (n=10) as measured on day 21 by flowcytometry following staining with anti-human CD45 antibody. (I) Relative number of hematopoietic mouse cells in the blood of untreated control mice and mice treated with BKT300. (J) Average percentage of human MV4–11 AML cells in the bone marrow (BM) of untreated control mice and BKT300-treated mice. (K) Relative number of mouse cells in the BM of untreated control mice and BKT300-treated mice. (L) Representative flow cytometry data of BM cells following staining with anti-human CD45 antibody, from untreated control mice and mice treated with BKT300, as described in panel J. Error bars represent mean ± SEM. Data are from two independent experiments. * p < 0.05, Student’s t-test.

BKT300 was initially tested in the U937 AML xenograft model, where early treatment, starting 3 days post-cell injection, resulted in 98% tumor growth inhibition, with 6/7 treated mice exhibiting complete tumor growth inhibition by day 11 (Fig. 7A). Dose-escalation studies found 2.5 mg BKT300 to be the most effective and well-tolerated dose (Fig. 7B). BKT300 treatment led to extensive cell death in the tumor tissue, as confirmed by the TUNEL assay, which showed BKT300-induced apoptosis of tumor cells (Fig. 7C). Immunohistochemical staining indicated that PRC1 expression was elevated in U937 tumors and remained unaffected by BKT300 treatment, while the treatment decreased CDC25C and increased pPRC1 T481 expression in the tumors (Fig. 7C). In addition to inhibiting U937 tumor growth in mice, BKT300 also induced tumor regression when administered starting 10 days post-cell injection, achieving 95.9% tumor growth inhibition (TGI) and 89.4% tumor regression (TR) by day 17 (Fig. 7D). No effect on body weight was observed in any of the treated mice. Additionally, BKT300 selectively targeted leukemic cells without affecting hematopoiesis or blood chemistry (Fig. 7E–F). Furthermore, safety studies across multiple animal models, including rats and non-human primates, confirmed that BKT300 had no adverse effects on hematopoiesis or biochemical parameters (data not shown).

In the IV model of MV4–11 FLT3 AML, which mimics human AML pathophysiology, BKT300 significantly reduced the percentage of AML cells in both the blood (74.8% inhibition, Fig. 7H) and BM (72% inhibition, Fig. 7J), while increasing the number of normal mouse cells in the BM (2-fold increase) and blood (7-fold increase), compared to control mice (Fig. 7I, 7K).

## Discussion

AML is a heterogeneous disease characterized by the uncontrolled proliferation of myeloid blast cells in BM. A critical challenge in treating AML lies in its complexity, driven by its heterogeneity and the genetic mutations and dysregulated signaling pathways that promote cell survival and resistance to treatment (36). Despite the fact that cell cycle inhibitors represent a promising class of therapeutic agents for AML (4), the specific role of PRC1 in this disease remains unexplored. This study highlighted the therapeutic potential of BKT300, a selective inhibitor of PRC1, for AML, particularly in cases with elevated PRC1 expression. BKT300 specifically inhibited PRC1, disrupting the organization and formation of microtubules, resulting in G2/M cell cycle arrest, mitotic catastrophe and caspase 3-dependent apoptosis. By preventing the dephosphorylation of PRC1 at Thr 481, BKT300 stabilizes the protein in a phosphorylated state, which inhibits its interaction with critical regulatory molecules such as PLK1. Prevention of PRC1-PLK1 complexation prevents proper cell division and migration, key events in cancer cell proliferation (14, 37, 38).

CDKs, particularly CDK1, play a key role in regulating microtubule dynamics during cell division by phosphorylating microtubule-associated proteins, tubulin, and motor proteins. They also modulate microtubule-destabilizing factors and localizing to mitotic structures (40, 41) .This regulation ensures proper spindle formation and chromosome segregation during mitosis. PRC1 is a substrate of CDK1 (10), and its phosphorylation is crucial for organizing antiparallel microtubules at the spindle midzone during cytokinesis. CDK1-mediated phosphorylation inhibits PRC1 activity until anaphase onset, preventing premature dimerization (10, 43). Specifically, PRC1 interacts with CDKs to regulate G2/M phase transition, a critical checkpoint for cell proliferation (10). Its role in regulation of microtubule dynamics during cell division and its influence on the cell cycle have been well-documented in other cancers (39). By modulating the expression of key cell cycle regulators, including p21 and CDC25C, BKT300 not only inhibits cell cycle progression but also promotes cell death in AML cells. Modulating the expression of key cell cycle regulators can promote cell death by disrupting the balance between proliferation and apoptosis, often through mechanisms such as p53 activation (44), CDK modulation (45), and alteration of pro- and anti-apoptotic gene expression (46).

CDC25C is a phosphatase that activates CDK1, driving cells into mitosis (47). By downregulating CDC25C expression, BKT300 disrupts the mitotic progression of AML cells, by inducing G2/M arrest and apoptosis. Dysregulation of CDC25C is associated with poor prognosis and is often linked to cancer progression (48, 49, 50, 51). Furthermore, members of the CDC25 family, including CDC25C, are implicated in the development of various human malignancies, including AML, rendering CDC25 inhibition a promising anticancer strategy for AML (52).

In AML, mutations in key genes, such as TP53, FLT3, NRAS, KMT2A and PTEN, are frequently observed and are often associated with poor prognosis, therapeutic resistance and distinct molecular profiles, which complicate disease management (36). Notably, BKT300 was effective across various cell lines with a range of mutations, including TP53, FLT3-ITD, NRAS, PTEN, and KMT2A, underscoring its broad therapeutic potential in targeting diverse genetic alterations in AML. Of particular mention is cells bearing TP53 mutations, which have been linked to aggressive disease, resistance to chemotherapy and short overall survival in AML patients (53, 54). PRC1 has been identified as a p53 target gene, revealing a novel function of p53 in regulating cytokinesis by controlling PRC1 transcription (22) Dysregulation of this pathway in TP53-mutant AML leads to PRC1 upregulation, which may enhance cancer cell survival and proliferation. By directly targeting PRC1, BKT300 offers a potential strategy for counteracting the resistance mechanisms associated with TP53-mutant AML.

BKT300 demonstrated significant efficacy in FLT3-internal tandem duplication (ITD)-expressing cell lines, such as MV4–11 and Molm14, inducing apoptosis independently of FLT3 mutation expression. Altered cell-cycle regulation has been reported in FLT3-ITD-positive AML (55, 56), yet the specific mechanisms by which FLT3 influences cell-cycle dynamics have remained unclear. FLT3 mutations, particularly FLT3-ITDs, are additional key drivers of AML progression. FLT3-ITD mutations lead to constitutive activation of the FLT3 receptor tyrosine kinase, promoting aberrant cell proliferation and survival (57, 58). Aberrant phosphorylation of CDC25C-T48 was identified in FLT3-ITD-mutated cells and CDC25C was demonstrated to be a downstream target of the mutated FLT3-ITD tyrosine kinase, affecting cell-cycle regulation in AML (55), and highlighting the role of this phosphatase in regulating the G2/M transition and promoting cell cycle progression. Moreover, PRC1 expression was observed to be elevated in bone marrow compared to peripheral blood in AML patients, highlighting its critical role in the progression of AML within the bone marrow microenvironment. This finding underscores the potential of BKT300 as a targeted therapeutic option for AML

In the AML xenograft mouse models, BKT300 effectively inhibited tumor growth and induced regression, without inducing significant side effects, highlighting its potential as a standalone therapeutic agent. Safety studies in multiple animal models, including mice, rats and non-human primates, confirmed that BKT300 does not adversely impact hematopoiesis or biochemistry, even at high doses.

In summary, this study demonstrated the promising therapeutic potential of BKT300, a first-in-class PRC1 inhibitor, for AML, particularly in cases with elevated PRC1 expression. By targeting PRC1, BKT300 disrupts cell cycle progression, inducing G2/M arrest and apoptosis. Its efficacy across lines with various genetic mutations and in preclinical models, along with its favorable safety profile, warrant its further development as a targeted therapy for AML. Additionally, combination therapy with other targeted agents or chemotherapies could enhance its effectiveness, particularly in overcoming resistance mechanisms in AML and other cancers.
